# The Anoikis Effector Bit1 Inhibits EMT through Attenuation of TLE1-Mediated Repression of E-Cadherin in Lung Cancer Cells

**DOI:** 10.1371/journal.pone.0163228

**Published:** 2016-09-21

**Authors:** Xin Yao, Tri Pham, Brandi Temple, Selena Gray, Cornita Cannon, Renwei Chen, Asim B. Abdel-Mageed, Hector Biliran

**Affiliations:** 1 Department of Biological and Public Health Sciences, Xavier University of Louisiana, New Orleans, Louisiana, United States of America; 2 Center for Bioengineering, University of California Santa Barbara, Santa Barbara, California, United States of America; 3 Tulane Cancer Center, Tulane University School of Medicine, New Orleans, Louisiana, United States of America; University of South Alabama Mitchell Cancer Institute, UNITED STATES

## Abstract

The mitochondrial Bcl-2 inhibitor of transcription 1 (Bit1) protein is part of an anoikis-regulating pathway that is selectively dependent on integrins. We previously demonstrated that the caspase-independent apoptotic effector Bit1 exerts tumor suppressive function in lung cancer in part by inhibiting anoikis resistance and anchorage-independent growth *in vitro* and tumorigenicity *in vivo*. Herein we show a novel function of Bit1 as an inhibitor cell migration and epithelial–mesenchymal transition (EMT) in the human lung adenocarcinoma A549 cell line. Suppression of endogenous Bit1 expression via siRNA and shRNA strategies promoted mesenchymal phenotypes, including enhanced fibroblastoid morphology and cell migratory potential with concomitant downregulation of the epithelial marker E-cadherin expression. Conversely, ectopic Bit1 expression in A549 cells promoted epithelial transition characterized by cuboidal-like epithelial cell phenotype, reduced cell motility, and upregulated E-cadherin expression. Specific downregulation of E-cadherin in Bit1-transfected cells was sufficient to block Bit1-mediated inhibition of cell motility while forced expression of E-cadherin alone attenuated the enhanced migration of Bit1 knockdown cells, indicating that E-cadherin is a downstream target of Bit1 in regulating cell motility. Furthermore, quantitative real-time PCR and reporter analyses revealed that Bit1 upregulates E-cadherin expression at the transcriptional level through the transcriptional regulator Amino-terminal Enhancer of Split (AES) protein. Importantly, the Bit1/AES pathway induction of E-cadherin expression involves inhibition of the TLE1-mediated repression of E-cadherin, by decreasing TLE1 corepressor occupancy at the E-cadherin promoter as revealed by chromatin immunoprecipitation assays. Consistent with its EMT inhibitory function, exogenous Bit1 expression significantly suppressed the formation of lung metastases of A549 cells in an *in vivo* experimental metastasis model. Taken together, our studies indicate Bit1 is an inhibitor of EMT and metastasis in lung cancer and hence can serve as a molecular target in curbing lung cancer aggressiveness.

## Introduction

Bit1 is a mitochondrial protein that is part of apoptosis pathway, which is uniquely regulated by integrin-mediated cell attachment. Following loss of cell attachment, Bit1 is released to the cytosol and interacts with the transcriptional regulator Amino Enhancer slip (AES) protein to induce a caspase-independent form of apoptosis [1]. While other anti-apoptotic factors such as Bcl-2, Bcl-xL, phosphatidylinositol 3-kinase, and Akt are unable to block the Bit1 apoptosis pathway, integrin-mediated cell attachment is the only upstream treatment that can suppress apoptosis induced by cytosolic Bit1. Hence, Bit1 may play a special role in detachment-induced apoptosis termed as anoikis by guarding the anchorage dependency of epithelial cells. In addition to integrin-mediated cell attachment, the groucho TLE1 corepressor protein which exhibits survival function in several cellular models [2–4], protects cells from Bit1 apoptosis.

The molecular mechanism of Bit1-mediated apoptosis has started to be unravelled. Forced expression of cytoplasmic Bit1 causes apoptosis in cells that express AES but not in the AES-null cell line. Further, AES potently induces apoptosis in cells that express Bit1. Importantly, the abundance of the Bit1-AES complex dictates the level of Bit1 apoptosis function. In line with the Bit1/AES complex as the apoptogenic factor, the integrin-mediated cell attachment and TLE1 corepressor protein block Bit1 apoptosis by inhibiting the formation of this complex [1]. Our collective data to date indicate that Bit1 through its functional interaction with AES switches off the survival promoting gene-transcription program mediated by TLE1 [5–7]. Consistent with the TLE1 nuclear pathway as a downstream target of Bit1, forced expression of cytoplasmic localized Bit1 or its cell death domain (CDD) induces significant re-localization of nuclear TLE1 to the cytoplasm in an AES dependent manner. Furthermore, exogenous expression of nuclear TLE1 effectively counteracts Bit1 apoptosis. Characterization of the TLE1 transcriptional pathway and its regulation by the Bit1/AES axis is currently under investigation.

Due to its independence from caspase activity, the Bit1 cell death pathway may represent as a unique caspase-independent anoikis mechanism in malignant cells and hence can serve as an important therapeutic target to abolish anoikis resistance particularly in caspase-deficient tumor cells. Since anoikis resistance is a hallmark of transformation and tumorigenesis, cancer cells may bypass this pathway to become anchorage independent and acquire tumorigenic phenotype [8]. Recently, we showed that the Bit1 pathway is functionally suppressed in Non-Small Cell Lung Carcinoma (NSCLC) as evidenced by the selective downregulation of Bit1 expression and upregulation of the Bit1 inhibitor TLE1 in advanced human lung tumors as compared to normal human lung tissues [9]. Importantly, targeted mitochondrial Bit1 expression in the caspase-deficient human NSCLC A549 cells attenuated their anoikis resistance and anchorage-independent growth *in vitro*. Conversely, stable downregulation of endogenous Bit1 in these cells conferred enhanced anoikis resistance, anchorage-independent growth potential, and tumorigenicity *in vivo* [9]. These collective data indicate a tumor-suppressive function of Bit1 in NSCLC.

In addition to promoting tumorigenesis, anoikis resistance is a determinant of tumor aggressiveness and metastasis [10]. Indeed, we have found that downregulation of endogenous Bit1 expression in the human breast cancer MCF7 and mouse melanoma B16F1 cell lines results in enhanced metastasis *in vivo* [11]. Furthermore, the exogenous expression of mitochondrial Bit1 in the highly aggressive melanoma B16F10 cells inhibits their metastatic potential [11]. These data show that Bit1 functions as a metastasis suppressor. To date, the molecular mechanism underlying Bit1 metastasis suppression has not been elucidated. However, it may involve inhibition of the extracellular signal-regulated kinase 1/2 (Erk1/2) survival signaling pathway, whose activity has been associated with cancer aggressiveness and metastatic potential. Bit1 inhibits Erk activation through induction of Erk-specific phosphatases, and inhibition of Erk activity contributes to Bit1 anoikis function [6]. However, the precise role of the Erk pathway in Bit1 metastasis suppression remains to be examined.

The ability of Bit1 to suppress metastasis may not be limited to its anoikis function [11]. Metastasis is a complex multi-step process involving tumor cell invasion of the underlying extracellular matrix at the primary site, intravasation into nearby circulatory and/or lymphatic system, and extravasation to lodge into the secondary sites [12]. Interestingly, downregulation of Bit1 expression in tumor cells conferred enhanced cellular migratory function and mesenchymal phenotype and was associated with loss of E-cadherin and upregulation of N-cadherin expression [11]. Although these phenotypic and molecular changes are consistent with epithelial-mesenchymal transition (EMT), a determinant of metastatic progression, the exact role of Bit1 in the regulation of EMT has not been fully investigated. Here, we show that Bit1 functions as an inhibitor of cell motility and EMT in lung cancer cells by upregulating the epithelial marker E-cadherin expression through inhibition of the TLE1 corepressor activity.

## Materials and Methods

### Cell culture and transfection assays

The human lung adenocarcinoma cell lines A549 and H460 from American Type Culture Collection (ATCC) were cultured in Dulbecco’s modified Eagle’s medium (DMEM) with glutamine containing 10% fetal bovine serum, penicillin, and streptomycin. The human bronchial epithelial cell line BEAS-2B was purchased from ATCC (ATCC CRL-9609) and cultured in BEGM Bronchial Epithelial Cell Growth Medium (Lonza). Transient transfection assays were carried out with lipofectamine 2000 (Invitrogen) in OPTI-MEM (Invitrogen) according to the manufacturer’s protocol with cells plated 18 hr before transfection and the total amount of plasmid used per transfection normalized with the corresponding empty vector construct [9]. The stable A549 Bit1 shRNA and control shRNA pool of cells were generated via transfection with pRS vector containing the short hairpin RNA against Bit1 (Origene) or the non-targeting scrambled shRNA (Origene) and selection with 1 μg/ml puromycin (Invitrogen). Individual puromycin-resistant clones were screened for Bit1 downregulation by immunoblotting using a specific antibody to Bit1 [9]. Similarly, the control shRNA and Bit1 shRNA BEAS-2B pool of cells were generated via transfection with pRS vector containing the short hairpin RNA against Bit1 (Origene) or the non-targeting scrambled shRNA (Origene) and selection with 0.5 μg/ml puromycin (Invitrogen). In both A549 and BEAS-2B parental lines, two Bit1 shRNA knockdown-positive clones and two control shRNA clones were pooled for further characterization. The control-GFP and GFP-TLE1 A549 pools of cells were generated by transduction with the lentiviral GFP-TLE1 or the empty control GFP construct (Open Biosystems, Huntsville, AL) [13].

### Chemical reagents, antibodies, and plasmids

The mouse monoclonal anti-myc, anti-FLAG, anti-GFP, and anti-B-actin antibodies were obtained from Sigma (St. Louis, MO), while the anti-E-cadherin and anti-vimentin antibodies were purchased from BD Biosciences (San Diego, CA). The mouse polyclonal anti-TLE1 antibody was obtained from Abcam (Cambridge, MA) while the anti-Zeb1 antibody was purchased from Santa Cruz Biotechnology, Santa Cruz, CA). The FLAG-AES and the various plasmid constructs encoding for Bit1 were generated as described previously [9]. The GFP-TLE1 and the full length E-cadherin encoding plasmids were obtained from Origene (Rockville, MD).

### siRNA transfection

Two previously validated independent non-overlapping specific Bit1 siRNAs (#1,AGACCUAAUUGACAAAGUCAC and #2, GAUACUGAAAGUGAAGCAAGC) were obtained from Invitrogen (Carlsbad, CA). The specific AES and E-cadherin siRNAs as well as the control, non-targeting siRNAs were also acquired from Invitrogen (Carlsbad, CA). The Zeb1 siRNAs were purchased from Santa Cruz Biotechnology (Santa Cruz, CA). As described previously [9], A549 cells (2 x 105) were transfected with 25 μM of each siRNA pool with the use of Lipofectamine RNAiMAX transfection reagent (Invitrogen). 48 hrs post-transfection, cells were harvested and subjected to immunoblotting, migration, real-time PCR, or reporter assays as described below. In some experiments, siRNA treated cells are subjected to plasmid transfection 24 hr after the siRNA treatment as described above.

### Analysis of cell migration and growth assay

The migratory ability of cells was quantified with the use of wound closure and Boyden chamber cell migration assays as described previously [13]. Wound closure experiments were performed by scarring cell monolayers with a sterile micropipette tip. Following 16 h of incubation, three defined areas were monitored and photographed (magnification, X100). In Boyden chamber migration assays, 2.5x10^4^ cells/well were seeded in the top chambers of a 24-well plate equipped with 8-μm pore-size micropore polycarbonate membrane filter (BD Biosciences). The lower chambers were filled with DMEM containing 10% FBS as a chemoattractant. Following 20h of incubation at 37°C, cells on the upper surface were carefully removed with a cotton swab, and the membranes were fixed 10% phospho-buffered formalin, permeabilized with 100% ice-cold methanol, and stained with 0.1% crystal violet in 20% methanol. The number of cells on the lower membrane was counted [13]. The wound closure and Boyden chamber assays were performed at least three times. Concurrently, we also assessed cell growth within the migration time. As described previously [9], anchorage-dependent growth was determined by the MTT assay. Briefly, cells were plated in a 96-well plate and the number of metabolically active cells at each indicated time was measured by adding ten microliters of the MTT reagent at 5 mg/ml (Sigma). Following incubation at 37°C, 5% CO2 for 3h, the resulting MTT precipitate was dissolved in 100 ul of a 50% MeOH-50% DMSO solution and subjected to a 550 nm absorbance reading via a microplate reader (BioTek Instruments).

### Cell aggregation assay

Cells were detached by treatment with trypsin (0.05%)-EDTA (0.53 mM) and suspended in Ca2+ free DMEM at 1x10^6^ cells/ml in polystyrene tubes. The tubes were then incubated on a rotating platform (10 rpm) at 37°C for 2 hours. The cell aggregation was viewed microscopically and photographed. The cell aggregation assay was repeated at least three times.

### Protein preparation and Western blotting assays

Protein preparation and immunoblotting were performed as described previously [9]. Briefly, cells were harvested by adding ice-cold NP-40 lysis buffer (1% NP-40; 20mM Tris-HCL [pH7.4]; 150 mM NaCl; 10% glycerol, 2 mM sodium vanadate; 1 mM henylmethylsulfonyl fluoride; 10 μg/ml leupeptine; and 5 μg/ml aprotinin) and incubated at 4°C for 20 min. For western blot analysis, equal amounts of proteins were resolved on 4–20% gradient Tris-glycine gels (Invitrogen) and electrophoretically transferred to nitrocellulose membrane. The membranes were incubated with primary antibodies at 37°C at room temperature followed by secondary antibodies conjugated with horseradish peroxidase. Membranes were developed using the ECL detection system.

### RNA extraction and Quantitative Real-Time PCR

Total RNA was extracted from 5.0x10^6^ cultured cells using the RNeasy kit (Qiagen) and quantified by spectrophotometry (NanoDrop 8000, Thermo Scientific) [13]. The RNA was subjected to a one step real-time RT-PCR using the iTaq Universal SYBR Green One-Step Kit (Bio-Rad) and cDNA quantification by real-time PCR on the BIO-RAD iQ5 Multicolor Real-Time PCR Detection System using the human E-cadherin (forward primer: AGGCTAGAGGGTCACCGCGTC and reverse primer: GCTTTGCAGTTCCGACGCCAC). The human GAPDH primers (forward primer: CCCACTCCTCCACCTTTGAC and reverse primer: TTGCTGTAGCCAAATTCGTTGT) were used for control.

### Luciferase reporter assays

The cell based reporter luciferase analysis was performed as described previously [13]. Briefly, cells were cotransfected with the E-cadherin luciferase promoter-reporter construct (SwitchGear Genomics) and the GAPDH luciferase promoter-reporter vector (SwitchGear Genomics) as an internal control. Twenty four hour post-transfection, the cells were subjected to a luciferase assay (SwitchGear Genomics’ LightSwitch Luciferase Assay System) per the manufacturer’s instructions. Luciferase activity was normalized and expressed relative to GAPDH luciferase activity. In certain experiments, cells transfected with plasmid or siRNAs were allowed to incubate for 24h prior to reporter luciferase assay (SwitchGear Genomics’ LightSwitch Luciferase Assay System) as described above. The promoter luciferase experiment was performed at least three times.

### ChIP assay

As described previously [13], cells were crosslinked with 1% formaldehyde and processed using the EpiSeeker ChIP Kit—One Step (Abcam). The resulting chromatin fragments were immunoprecipitated with the anti-TLE1 antibody-ChIP Grade (Abcam), anti-Acetyl-Histone H3-ChIP grade (anti-Ac-H3) (EMD MILLIPORE), or non-specific IgG (Santa Cruz Biotechnology). Subsequent downstream steps were conducted following the protocol from the EpiSeeker ChIP Kit (Abcam). The PCRs were conducted using the E-cadherin primers (5'-CCCACCACGTACAAGGGTC-3`(sense), 5'-ATGCCATCGTTGTTCACTGGA-3`(antisense)) with the following program: 45 cycles at 95°C for 30 s, 56°C for 30s, and 72°C for 30s as described previously [13]. The amplified E-cadherin DNA promoter fragment was separated on 1.5% agarose gel and visualized with ethidium bromide. The purified DNA samples were used as an input for PCR reactions. The PCR product was then subjected to densitometric quantification using QuantityOne software (Bio-Rad). Fold enrichment was determined and expressed as +TLE1-specific antibody over IgG or +Acetyl-Histone H3-specific antibody over IgG. The ChIP experiments were repeated at least three times.

### *In vivo* metastasis assays

All procedures were done according to protocols approved by the Institutional Committee for Use and Care of Laboratory Animals of Xavier University of Louisiana Institutional Animal Care and Use Committee (IACUC, Approval Number 060911-001BI). The Animal Care Facility of Xavier University of Louisiana ensures the best husbandry and environment for maintaining and caring of laboratory animals and complies with all applicable governmental guidelines. Groups of eight female athymic nude mice (BALB/c) were used for the experimental metastasis assay. The A549 derived control and Bit1 mito cells (2.0 x10^6^) or the control shRNA and Bit1 shRNA cells (1.0 x10^6^) were injected via the tail vein. Following injection, mice were observed at least three times weekly for clinical signs associated with tumor progression including decreased food/water intake, weight loss, respiratory difficulty, neurological signs, and restricted mobility. Animals found to be experiencing unrelievable pain and distress were subject to euthanasia. Overdose of inhalant anesthetic followed by cervical dislocation was the method used for euthanasia. Seven weeks (for control and Bit1 mito cells) and six weeks (for control shRNA and Bit1 shRNA cells) post-injection, mice were sacrificed and the lungs were excised and examined for presence of metastatic surface nodules and subsequently fixed in 4% PFA overnight, cryoprotected in 30% sucrose in PBS and frozen in OCT embedding media (Tissue Tek), as previously described [11]. Serial sections of the lungs were stained with Hematoxylin and Eosin (H&E), and the number of microscopic colonies was counted under a light microscope.

### Statistical Analysis

Data are presented as means ±S.E.. For Western blotting, migration, real-time, reporter, and ChIP assays, experiments were performed at least three times. Statistical differences between groups were established at a P value < 0.05 using the two-tailed Student’s *t*-test. All calculations were done using the NCSS statistical software (NCSS, Kasville, UT).

## Results

### Downregulation of Bit1 expression induces EMT in A549 cells and inhibits the epithelial phenotype of BEAS-2B cells

**Fig 1:**
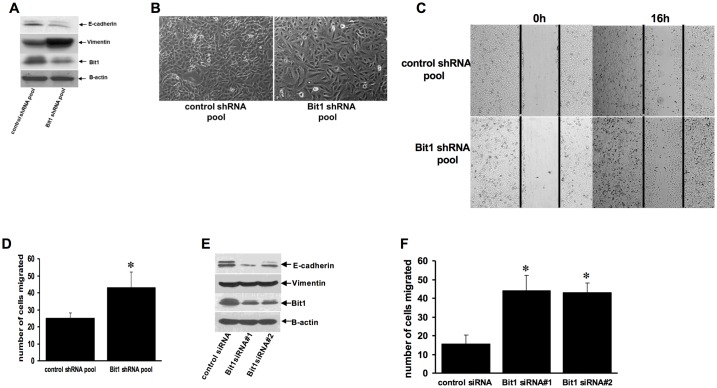
Knockdown of Bit1 expression promotes EMT in A549 cells. A. Stable control shRNA and Bit1 shRNA pool of cells derived from the A549 cell line were subjected to total cell lysate isolation, SDS-PAGE, and immunoblotting against specific antibodies to E-cadherin, vimentin, Bit1, and B-actin. In B, C, and D, the control shRNA and Bit1 shRNA cells were subjected to phase contrast microscopy (100x magnification) under normal culture conditions (B), wound closure (C), and Boyden chamber migration (D) assays. E and F. A549 cells were transfected with control or Bit1 specific siRNAs, and 48 h post-transfection, cells were harvested and subjected to immunoblotting against the indicated antibodies (E) and Boyden chamber migration assay (F). In D and F, results are representative of three independent experiments, *p<0.05 as compared with the control cells (Student’s t test).

We have previously observed that suppression of endogenous Bit1 expression in the human cervical cancer Hela cells resulted in enhanced spindle shape-like morphology and migratory capacity and molecular changes consistent with EMT [11]. To investigate the role of Bit1 in EMT, we examined the effect of altering Bit1 expression on the EMT phenotype of the human lung adenocarcinoma A549 cell line. We first examined any notable EMT related morphological changes in these cells following stable downregulation of the endogenous Bit1 expression. A pool of stable Bit1 knockdown A549 cells was generated using the shRNA strategy [9]. The Bit1 shRNA pool exhibited 70–80% downregulation of Bit1 expression as compared to control shRNA pool of cells (Fig 1A). The Bit1 shRNA pool of cells displayed a flatter, stretched fibroblast-like appearance (Fig 1B) and enhanced cell motility (Fig 1C and 1D) relative to control shRNA cells. The Bit1 shRNA pool nearly closed the wound 16h post-initiation of a wound repair assay, which remained open in control shRNA cells (Fig 1C). The increased motility of Bit1 shRNA cells was about 2 fold as compared to control shRNA cells as determined by the Boyden chamber migration assay (Fig 1D). It is noteworthy that the control shRNA and Bit1shRNA cells exhibit similar growth kinetics within the migration time frame, indicating that the observed enhanced migration of Bit1 knockdown cells is not likely attributable to changes in cell growth (S1 Fig). We then examined whether these observed EMT phenotypes were associated with changes on known molecular markers of EMT. As assessed by immunoblotting (Fig 1A), the stable Bit1 shRNA A549 cells showed significantly reduced levels of the epithelial marker E-cadherin with concomitant increased levels of the mesenchymal marker vimentin as compared to the control shRNA cells. To confirm these results, we also performed transient knockdown of endogenous Bit1 expression in A549 cells with the use of previously validated two specific Bit1 siRNAs (Fig 1E). Targeted reduction of Bit1 significantly enhanced cell motility (Fig 1F, S1B Fig) and suppressed E-cadherin expression (Fig 1E). Interestingly, acute ablation of Bit1 expression did not significantly alter the expression of vimentin, a late stage EMT marker, suggesting that E-cadherin is likely an immediate target of Bit1 in regulating EMT.

**Fig 2:**
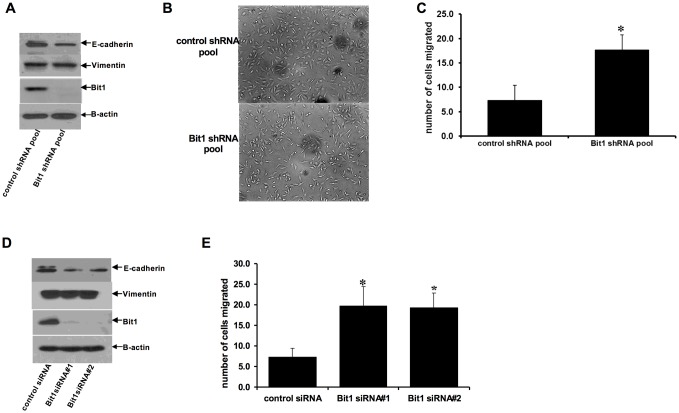
Suppression of Bit1 expression attenuates the epithelial phenotype of BEAS-2B cells. Stable control shRNA and Bit1 shRNA pool of BEAS-2B cells were subjected to immunoblotting with the indicated antibodies (A), phase contrast microscopy (B), and Boyden chamber migration assay (C). D and E. BEAS-2B cells were treated with control or Bit1 specific siRNAs, and 48h later, cells were subjected to immunoblotting against the indicated antibodies (D) and Boyden chamber migration assay (E). In C and E, results are representative of three independent experiments, *p<0.05 as compared with the control cells (Student’s t test).

To further examine the role of Bit1 in EMT during lung carcinogenesis, we investigated whether downregulation of endogenous Bit1 expression attenuates the epithelial phenotype of the immortalized, non-tumorigenic human bronchial epithelial cell line BEAS-2B. Stable knockdown of Bit1 expression in Bit1 shRNA BEAS-2B cells resulted in spindle shaped morphology with reduced cell-cell contact in monolayer culture, while the control shRNA BEAS-2B cells maintained their epithelial morphology (Fig 2A and 2B). Additionally, the stable Bit1shRNA BEAS-2B cells exhibited increased migration potential (Fig 2C) and reduced E-cadherin expression (Fig 2A). To corroborate these findings, the endogenous Bit1 expression in BEAS-2B was also transiently downregulated via the siRNA strategy (Fig 2D). The Bit1siRNA treated BEAS-2B cells exhibited enhanced migration (Fig 2E) and reduced E-cadherin expression (Fig 2D) as compared to control siRNA cells. It is noteworthy that stable and transient knockdown of Bit1 expression in BEAS-2B cells did not significantly alter their growth kinetics relative to control cells within the migration time frame (S1C and S1D Fig, indicating that the observed enhanced motility of Bit1 knockdown cells is not due to changes in survival. The observed minimal changes in vimentin expression in BEAS-2B following acute and chronic ablation of Bit1 further suggest that E-cadherin is likely an immediate target of Bit1 in regulating EMT. Collectively, these studies indicate that Bit1 functions to maintain the normal epithelial phenotype and its downregulation may promote EMT.

### Ectopic expression of Bit1 inhibits EMT and induces E-cadherin expression

**Fig 3:**
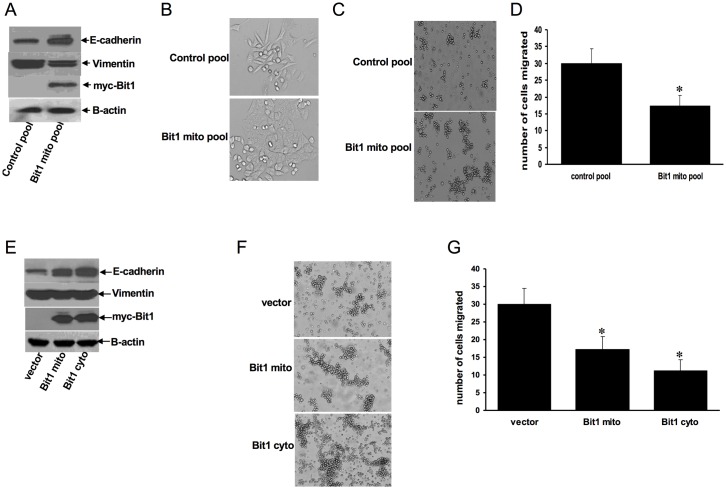
Bit1 induces E-cadherin expression and inhibits EMT. A. Stable control and mitochondrial Bit1 expressing (Bit1 mito) pool of A549 cells were subjected to immunoblotting with the indicated antibodies. B and C. Stable control and Bit1 mito pool of cells were subjected to phase contrast microscopy under normal culture conditions (B) and in suspension (C). D. Stable control and Bit1 mito pool of cells were subjected to Boyden chamber migration assay. E, F, and G. A549 cells were transfected with the C-terminally myc-tagged mitochondrial localized Bit1 (Bit1 mito), N-terminally tagged cytoplasmic localized Bit1 (Bit1 cyto) or empty vector construct. 24 h post-transfection, cells were subjected to immunoblotting against the indicated antibodies (E), phase contrast microscopy in suspension culture (F), and Boyden chamber assay (G). In D and G, three independent experiments were performed in triplicates, * indicates p<0.05 by Student’s t test as compared to control cells.

To further study the role of Bit1 in EMT, we investigated whether exogenous Bit1 could drive an epithelial phenotype in A549 cells. We stably expressed mitochondrial Bit1 in A549 cells via transfection of the full-length C-terminal myc tagged Bit1 construct, which expresses the Bit1 protein exclusively in the mitochondria (Bit1 mito) [9], or empty vector. The expression of the exogenous mitochondrial Bit1 protein in A549 cells was confirmed by immunoblotting (Fig 3A). In contrast to vector control pool of cells, which exhibited a spindly, elongated phenotype, the stable mitochondrial Bit1 cells showed a cobblestone and compact growth pattern in monolayer culture (Fig 3B). Further, the stable Bit1 mito cells showed increased aggregation in suspension (Fig 3C) and reduced motility (Fig 3D) as compared to vector control cells. Consistent with these epithelial phenotypes, the stable mitochondrial Bit1 cells displayed increased expression of E-cadherin and decreased expression of Vimentin (Fig 3A). To confirm these findings, we also transiently expressed exogenous Bit1 in A549 cells via transfection with the C-terminally myc tagged Bit1 (Bit1 mito), the N-terminally myc tagged Bit1, which targets Bit1 expression in the cytosol (Bit1 cyto) [9], or vector construct (Fig 3E). Ectopic Bit1 induced increased cell aggregation in suspension (Fig 3F) and decreased cell motility (Fig 3G) in A549 cells. The cytoplasmic Bit1 was particularly more effective in inducing these epithelial phenotypes. It is noteworthy that the growth rate between the control and Bit1-expressing cells during the migration assay was comparable (S1E Fig), suggesting that the observed inhibition of cell motility by exogenous Bit1 is not due to changes in cell growth. At the molecular level, acute expression of exogenous Bit1 increased the E-cadherin protein level with no significant effect on vimentin (Fig 3E), which is in line with the notion that E- cadherin is a direct target of Bit1 in regulating EMT. Collectively, these findings indicate that Bit1 drives an epithelial phenotype and reverses EMT in A549 cells.

### Bit1 regulates E-cadherin expression at the transcriptional level in an AES-dependent manner

**Fig 4:**
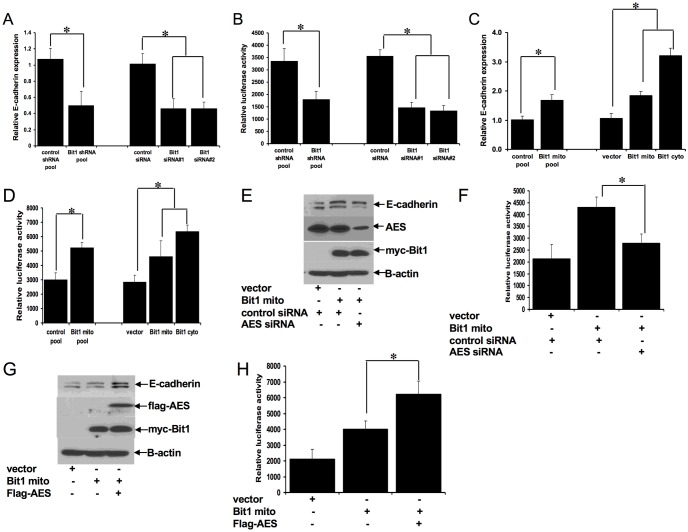
Bit1 regulates E-cadherin expression at the transcriptional level through the transcriptional regulator AES. A. and B. Stable control shRNA and Bit1 shRNA A549 cells as well as A549 cells treated with control or Bit1 siRNAs were subjected to total RNA isolation, reverse transcription, and real time PCR analysis using specific E-cadherin primers (A) and E-cadherin promoter luciferase activity assay (B) as described in materials and methods. C and D. Stable control and Bit1 mito A549 cells as well as A549 cells transiently transfected with vector, Bit1 mito, or Bit1 cyto construct were subjected to RNA isolation and Real time PCR analysis (C) to assess for E-cadherin mRNA levels and E-cadherin promoter luciferase assay (D) to quantify E-cadherin promoter activity. E. and F. A549 cells were treated with control or AES siRNAs, and 24 hr later cells were transfected with the C-terminally myc-tagged Bit1 (Bit mito) or empty vector construct as indicated. 24h after the plasmid transfection, cells were harvested and subjected to immunoblotting with the indicated antibodies (E) and E-cadherin promoter luciferase assay (F). G and H. A549 cells were transfected with the indicated construct. The amount of plasmid transfected into cells was normalized with the vector construct. 24h post transfection, cells were harvested and subjected to immunoblotting with the indicated antibodies (G) and E-cadherin promoter luciferase assay (H). In A, B, C, D, F, and H, three independent experiments were performed in triplicates, * indicates p<0.05 by Student’s t test.

An important hallmark and early marker of EMT is the loss of the tumor suppressor epithelial marker E-cadherin expression [14]. As a potential target of Bit1 in regulating EMT, we examined the mechanism of E-cadherin regulation by Bit1. Given that transcriptional control is an important mechanism in altering E-cadherin expression in solid tumors [15–16], we examined if Bit1 regulates E-cadherin expression at the transcriptional level via real-time PCR and promoter assays in A549 cells. In line with the observed reduction of E-cadherin protein, the Bit1 shRNA and siRNA treated A549 cells exhibited a significantly reduced level of E-cadherin mRNA transcript (Fig 4A) and reporter activity (Fig 4B) as compared to control shRNA and siRNA cells, respectively. Furthermore, exogenous Bit1 expressing A549 cells showed increased levels of E-cadherin transcript (Fig 4C) and reporter activity (Fig 4D). Consistent with our immunoblotting studies (Fig 3E), the cytoplasmic localized Bit1 was more effective in inducing E-cadherin expression (Fig 4C and 4D). Since the Bit1 anoikis function is dependent on the Groucho transcriptional regulator AES protein [1,7], we then examined the role of AES on Bit1 regulation of E-cadherin expression. As shown in Fig 4E and 4F, downregulation of AES via the siRNA strategy attenuated the induction of E-cadherin expression by Bit1. Conversely, exogenous AES potentiated the Bit1 induced E-cadherin expression (Fig 4G and 4H). Together, the results indicate that Bit1 induces E-cadherin expression at the transcriptional level in part through AES.

### Induction of E-cadherin is necessary for Bit1 inhibition of cell motility

**Fig 5:**
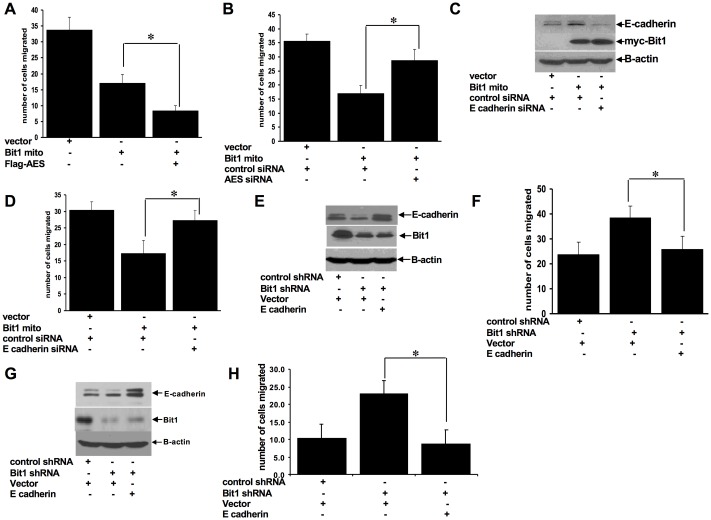
Induction of E-cadherin expression contributes to Bit1-mediated inhibition of cell motility. A. A549 cells were transfected with C-terminally tagged mitochondrial Bit1 (Bit1 mito) alone or together with FLAG-tagged AES construct. The amount of plasmid transfected into cells was normalized with the vector construct. 24h post transfection, cells were harvested and subjected to Boyden chamber migration assay. B. A549 cells were treated with control- or AES-siRNAs, and 24 h later cells were transfected with the vector or Bit1 mito construct as indicated. 24h after the plasmid transfection, cells were subjected to Boyden chamber migration assay. C. and D. A549 cells were treated with control- or E-cadherin-siRNAs, and 24 h later cells were transfected with the vector or Bit1 mito construct as indicated. 24h after the plasmid transfection, cells were harvested and subjected to immunoblotting with the indicated antibodies (C) and Boyden chamber migration assay (D). E. and F. Stable control shRNA and Bit1 shRNA A549 cells were transfected with the vector or E-cadherin construct as indicated. 24h post-transfection, cells were harvested and subjected to immunoblotting with the indicated antibodies (E) and Boyden chamber migration assay (F). G. and H. Stable control shRNA and Bit1 shRNA BEAS-2B cells were transfected with the vector or E-cadherin construct as indicated. 24h post-transfection, cells were harvested and subjected to immunoblotting with the indicated antibodies (G) and Boyden chamber migration assay (H). In A, B, D, F, and H, three independent experiments were performed in triplicates, * indicates p<0.05 by Student’s t test.

Based on our findings that Bit1 regulates E-cadherin expression, we hypothesize that E-cadherin is a downstream target of Bit1 in regulating EMT. To test this possibility, we examined whether E-cadherin expression is required in Bit1 inhibition of cell motility. Considering that AES potentiates the Bit1 induction of E-cadherin (Fig 4E–4H), we first examined the impact of AES on Bit1 regulation of cell motility. Exogenous AES increased the migration inhibitory activity of Bit1 (Fig 5A), while knockdown of endogenous AES expression attenuated the Bit1 inhibitory effect on cell migration (Fig 5B). To directly assess the role of E-cadherin in the Bit1 regulation of cell motility, Bit1 mito transfected A549 cells were treated with control or E-cadherin siRNAs and then subjected to a Boyden chamber migration assay (Fig 5C and 5D). Indeed, forced downregulation of E-cadherin expression in Bit1 transfected cells significantly attenuated the Bit1-induced inhibition of cell motility. To confirm these findings, we ectopically expressed E-cadherin in the Bit1 shRNA A549 cells. As shown in Fig 5E and 5F, exogenous E-cadherin diminished the enhanced motility of Bit1 shRNA cells as compared to control shRNA cells. Similar results were also found when ectopic E-cadherin was introduced in Bit1 shRNA BEAS-2B treated cells (Fig 5G and 5H). Taken together, these findings indicate that induction of E-cadherin expression is necessary for Bit1 to inhibit lung cancer cell motility and further suggest that E-cadherin is a downstream target of Bit1 in repressing EMT.

### Bit1 upregulates E-cadherin expression through inhibition of TLE1 corepressor function

**Fig 6:**
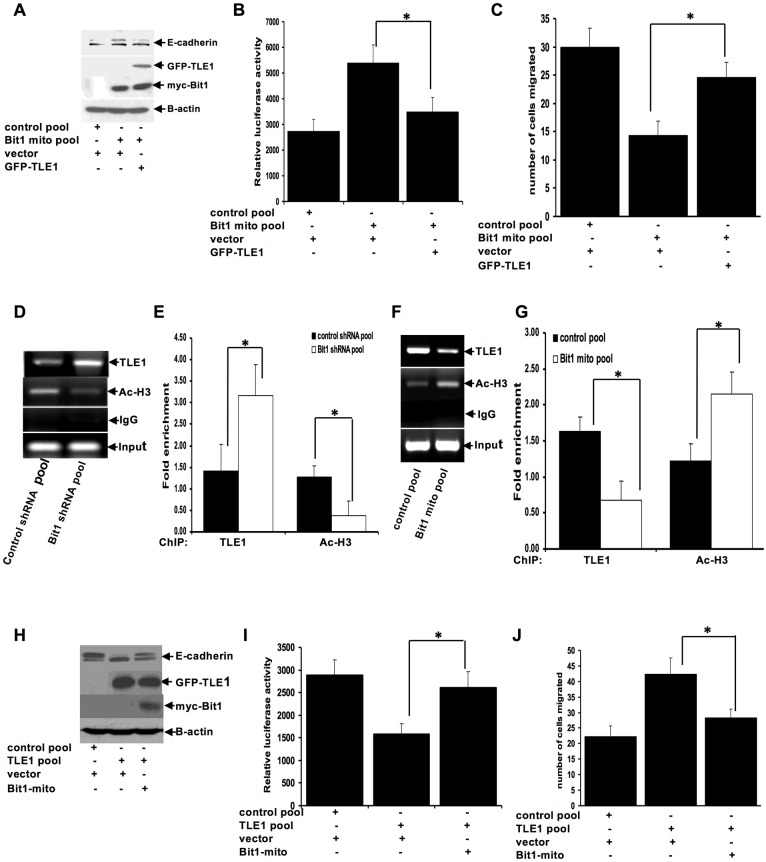
Bit1 negatively regulates TLE1-mediated E-cadherin repression. A., B. and C. Stable control and Bit1 mito A549 cells were transfected with the GFP-tagged TLE1 or vector construct. 24h post transfection, cells were harvested and subjected to immunoblotting with the indicated antibodies (A), E-cadherin promoter luciferase analysis (B), or Boyden chamber migration assay (C). D. and E. Stable control shRNA and Bit1 shRNA A549 cells were analysed by ChIP assay. Chromatin was precipitated using antibodies against TLE1, acetyl-histone H3 (Ac-H3), and control IgG as detailed in the materials. The E-cadherin promoter sequence was amplified by PCR and subjected to agarose gel electrophoresis (D). The ChIP experiments were repeated at least three times and a representative experiment is shown. Enrichment of the E-cadherin promoter fragment in TLE1-ChIP and acetyl-histone 3-ChIP over IgG-antibody in control shRNA and Bit1 shRNA cells is shown in E. F. and G. Stable control and Bit1 mito A549 cells were subjected to ChIP assay with antibodies against TLE1, acetyl-histone H3 (Ac-H3), and control IgG. The E-cadherin promoter fragment was amplified by PCR and subjected to agarose gel electrophoresis (F). Enrichment of the E-cadherin promoter fragment in TLE1-ChIP and acetyl-histone 3-ChIP over IgG-antibody in control and Bit1 mito cells is shown in G. H., I., and J. Control and TLE1 expressing pool of A549 cells were transfected with the Bit1 mito or empty vector construct, and 24h later cells were harvested and subjected to immunoblotting with the indicated antibodies (H), E-cadherin promoter luciferase analysis (I), and Boyden chamber migration assay (J). In B, C, E, G, I, and J, three independent experiments were performed in triplicates, * indicates p<0.05 by Student’s t test.

We have previously shown that Bit1 induces anoikis in part through inhibition of the survival promoting TLE1 transcriptional machinery [7]. In light of the recently identified novel EMT promoting function of the TLE1 corepressor [13], we then investigated whether TLE1 is a downstream target of Bit1 in regulating EMT. First, we characterized the components of the TLE1-medited E-cadherin repression by examining the role of known E-cadherin transcriptional repressors in this process. Based on the previous finding demonstrating the unique correlation of the transcription factor Zeb1 with loss of E-cadherin expression in human lung cancer cell lines [17], here we only examined the role of Zeb1 on the observed TLE1-mediated E-cadherin repression. As shown in S2A and S2B Fig, knockdown of Zeb1 attenuated the ability of TLE1 to repress E-cadherin, indicating that the TLE1-mediated E-cadherin repression is dependent on Zeb1. To address if TLE1-mediated E-cadherin repression is a target of Bit1 EMT inhibitory function, we then determined if TLE1 can block Bit1 inhibition of E-cadherin repression. As shown in Fig 6A–6C, the ectopic TLE1 attenuated Bit1 induced E-cadherin expression and inhibition of cell motility. Importantly, the ability of TLE1 to attenuate Bit1-induced upregulation of E-cadherin expression required Zeb1 (S3A and S3B Fig).

Based on the antagonistic effect of TLE1 on Bit1 regulation of EMT, we then examined if Bit1 impinges on TLE1 nuclear function. As an inducer of EMT, the TLE1 corepressor silences E-cadherin expression in part by recruiting the HDAC to the E-cadherin promoter [13]. To address the possibility that Bit1 may alter TLE1 localization on the E-cadherin promoter, ChIP assay with the specific antibody against TLE1 was performed in the stable Bit1 knockdown and control cells (Fig 6D and 6E). Consistent with our earlier results [13], endogenous TLE1 was found to interact with the E-cadherin promoter and this interaction was specific for TLE1 due to the absence of the E-cadherin promoter sequences in the control IgG immunoprecipitate. Importantly, the level of TLE1 in the E-cadherin promoter was significantly enhanced in the Bit1 knockdown cells as compared to control cells. In line with the HDAC recruitment function of TLE1, the observed increased TLE1 occupancy on the E-cadherin promoter in Bit1 knockdown cells was associated with a decreased level of acetylated histone H3 (Fig 6D and 6E). To further assess the effect of Bit1 on TLE1 occupancy at the E-cadherin promoter region, ChIP assay against the TLE1 antibody was performed on the stable control and mitochondrial Bit1 expressing cells. As shown in Fig 6F and 6G, Bit1 expressing cells exhibited significantly reduced TLE1 binding with a concomitant increase in acetylated histone on the E-cadherin promoter. In line with these results, exogenous Bit1 expression attenuated TLE1 induced suppression of E-cadherin expression and increase in cell migration (Fig 6H–6J). Taken together, these findings suggest that TLE1 is a downstream target of Bit1 in regulating E-cadherin expression and EMT.

### Bit1 inhibits metastasis of A549 lung cancer cells *in vivo*

**Fig 7:**
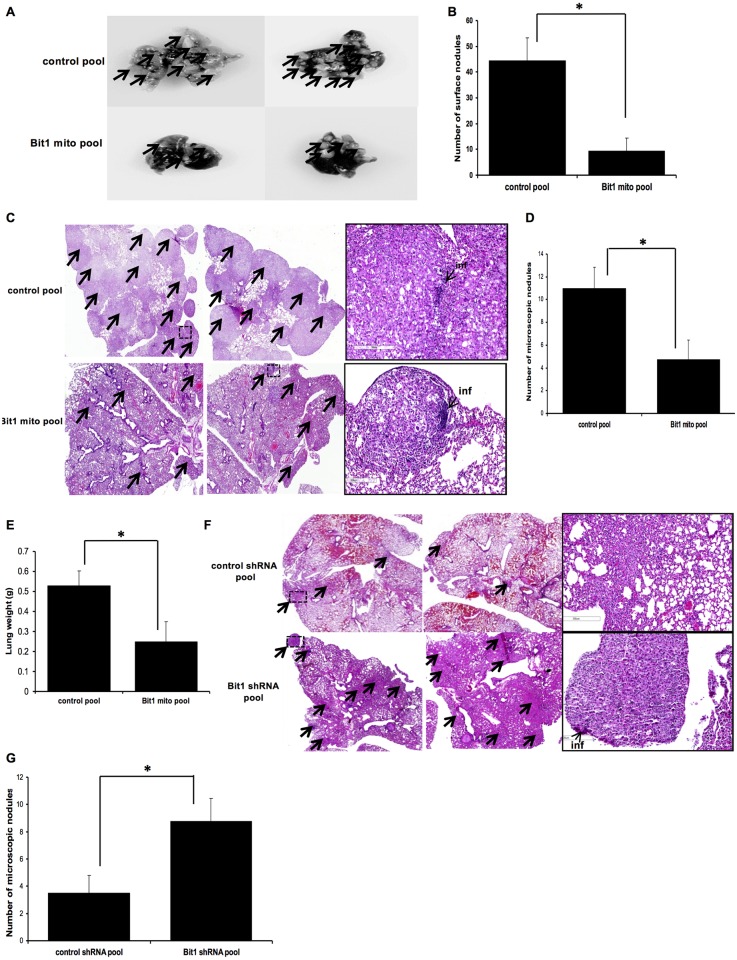
Bit1 inhibits tumor metastasis *in vivo*. A., B., C. D., and E. Stable control and Bit1 mito A549 cells were injected into the tail vein of nude mice. After seven weeks post-injection, the mice were sacrificed and the lungs were harvested and photographed (the representative lungs are shown in A) and the number of surface tumor nodules was counted (B). The lung samples were subjected to histological analysis. Representative H & E staining of lung tissue sections is shown in C. The number of microscopic tumor nodules was quantified in D. The total lung weight from the control and Bit1 mito group of mice was measured (E). F. and G. The control shRNA and Bit1 shRNA A549 cells were injected into the tail vein, and six weeks after injection mice were sacrificed and subjected to histological analysis. The representative H & E staining of the lung sections is shown in F. The number of microscopic tumor nodules in the lung sections was quantified (G). In C and F, boxed regions are shown at higher magnification showing inflammatory (inf) cells surrounding the tumor nodules. The arrows denote tumor nodules. In B, D, E, and G, * indicates p<0.05 by Student’s t test.

Since EMT is a critical determinant of tumor metastasis, we then examined the effect of Bit1 on metastatic ability of A549 cells. To this end, the propensity of control and mitochondrial Bit1 expressing cells to disseminate to lungs in an experimental metastasis assay was examined following injection of 2 x10^6^ cells into the tail vein of athymic nude mice. As shown in Fig 7A and 7B, lungs derived from mice injected with Bit1 expressing cells showed a significant decrease in the number of surface tumor nodules as compared to that of control cells. Histological examination of the isolated lungs further revealed a reduction in the number and size of metastatic nodules from mice inoculated with Bit1 cells (Fig 7C and 7D). Consistent with the reduced pulmonary metastatic colonization by the Bit1 cells, the total lung weight in mice that received injections of the Bit1 cells was significantly reduced relative to that of the control cells (Fig 7E). To ascertain these results, the control shRNA and Bit1 shRNA cells were also subjected to an experimental metastasis assay with 1 x10^6^ cells injected into the tail vein of nude mice. Indeed, the lungs of mice that received injections of the Bit1 knockdown cells showed an increase in the number of metastatic foci relative to the control cells (Fig 7F and 7G). Taken together, the findings indicate that Bit1 inhibited the metastatic behavior of A549 lung cancer cells *in vivo*.

## Discussion

Bit1 is a mitochondrial protein that is involved in an integrin-dependent apoptosis pathway [1]. Upon loss of cell attachment, Bit1 is released to the cytoplasm to induce a caspase independent form of cell death. Indeed, we have shown that ectopic Bit1 can enhance anoikis and inhibit the anchorage-independent growth of the caspase-resistant NSCLC A549 cells *in vitro*. Furthermore, downregulation of endogenous Bit1 expression in A549 cells resulted in enhanced tumorigenicity with a concomitant reduction in tumor cell apoptosis *in vivo*. Importantly, Bit1 expression was found to be selectively downregulated in various types of human NSCLC tumors as compared to normal lung tissues [9]. Although these collective data indicate a tumor suppressive role of Bit1 in NSCLC, the function of Bit1 in lung cancer motility and aggressiveness remain largely unknown.

Loss of endogenous Bit1 expression has been associated with enhanced migration and EMT-like phenotype in tumor cells [11]. However, the underlying mechanism of Bit1 regulation of cell motility and EMT has not been examined. Here, using the NSCLC A549 model system, we show that Bit1 functions as an inhibitor of cell migration and EMT in part by inducing E-cadherin expression. This finding was reinforced by the loss of epithelial phenotype and acquisition of fibroblastic mesenchymal morphology and enhanced migration potential in A549 and BEAS-2B Bit1-silenced cells. Strikingly, exogenous Bit1 expression induced A549 cells to undergo epithelial transformation characterized by cobblestone growth pattern in monolayer culture, increased multicellular aggregation in suspension, and decreased motility. Consistent with their epithelial phenotype, Bit1 expressing cells showed increased expression of the epithelial marker, E-cadherin. Thus, our current findings raise the exciting possibility that Bit1 exert its tumor suppressor function in lung cancer not only by inducing anoikis, but also by inhibiting EMT, a critical event in tumor aggressiveness and progression.

Loss or downregulation of E-cadherin adhesive protein is a hallmark of EMT, and as such, examination of the molecular mechanisms underlying regulation of E-cadherin expression has been the focus of many studies [14]. The expression of the epithelial marker E-cadherin is frequently lost in various types of cancer, and functional genetic studies have demonstrated a critical role of E-cadherin downregulation in promoting tumor aggressiveness and metastasis [14]. Indeed, inhibition of E-cadherin expression is a consequence of activation of oncogenes and silencing of tumor suppressor genes. Here, we show that E-cadherin is a target of the tumor suppressor Bit1, and the induction of E-cadherin expression in lung cancer cells is necessary for the Bit1 migration inhibitory function. In particular, forced downregulation of E-cadherin was sufficient to block Bit1 inhibition of cell motility, whereas the ectopic E-cadherin expression reversed the enhanced migration of Bit1 knockdown cells. While these data demonstrate E-cadherin as a critical target of Bit1 in inhibiting cell motility, future studies are required to elucidate the impact of E-cadherin targeting by Bit1 on other EMT phenotypic features. Interestingly, one of the hallmarks of EMT is acquisition of anoikis resistance by tumor cells [18]. Since Bit1 is a potent anoikis effector, it will be interesting to determine whether the observed induction of E-cadherin expression by Bit1 plays a role in its anoikis function. It is noteworthy that the ability of the neutrophic receptor tyrosine kinase TrkB to block anoikis and induce EMT has been linked to suppression of E-cadherin expression [19].

**Fig 8:**
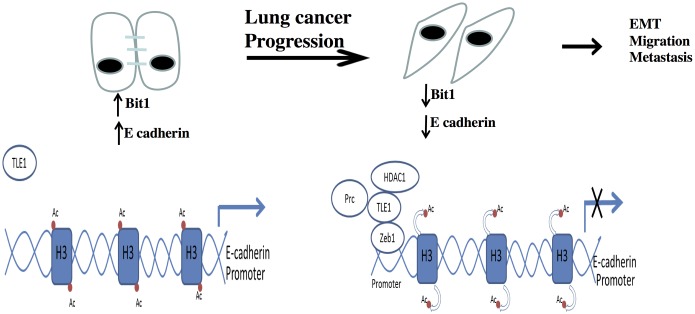
A model depicting the regulation of EMT and metastasis by Bit1 through inhibition of TLE1-mediated repression of E-cadherin expression. In normal bronchial epithelial cells, Bit1 upregulates E-cadherin expression and maintains epithelial phenotype by relieving E-cadherin repression through removal of TLE1 corepressor and its associated repressive chromatin remodelling enzymes such as HDAC1 at the E-cadherin promoter. During lung cancer progression, loss of Bit1 expression results in suppression of E-cadherin expression as a consequence of enhanced TLE1 occupancy, HDAC1 recruitment, and histone deacetylation at the E-cadherin promoter. The suppression of E-cadherin upon downregulation of Bit1 may promote EMT, migration, and metastasis. While our current data indicate that the Zeb1 transcription factor may in part underlie TLE1 recruitment to the E-cadherin promoter to repress transcription, additional components of the TLE1 corepressor machinery including other known E-cadherin repressors and chromatin remodelling Polycomb repressive complexes (Prc) remain to be identified.

A number of E-cadherin transcriptional repressors (EcTRs) including ZEB, Snail, and Twist have been identified and their expression in epithelial derived tumors is associated with increased EMT, aggressiveness, metastasis, and poor clinical outcome. EcTRs bind to E boxes domain in the E-cadherin promoter and recruit corepressor complexes containing chromatin modifying enzymes. In particular, SNAIL and ZEB repression of E-cadherin involves binding to the C-terminal binding protein (CtBP) corepressor, which in turn recruits histone deacetylase (HDACs) to alter the local chromatin structure [20,21]. We recently demonstrated that the Groucho TLE1 is a novel corepressor which represses E-cadherin in lung cancer cells, in part by recruiting HDAC1 to the E-cadherin promoter [13]. As demonstrated in this report (S2A and S2B Fig), the TLE1 recruitment to the E-cadherin promoter is to some extent dependent on Zeb1. In light of the complex interplay among known E-cadherin transcriptional repressors in the regulation of E-cadherin expression, we cannot exclude the possibility that other transcription factor(s) may also regulate that ability of TLE1 to repress E-cadherin expression. Hence, our future studies will be directed toward examining the potential role of other E-cadherin repressors including Snail and Slug on TLE1-induced E-cadherin repression. Furthermore, the present study indicates that Bit1 transcriptionally upregulates E-cadherin expression by inhibiting the TLE1 corepressor function. While exogenous Bit1 expression resulted in reduced TLE1 occupancy and increased histone acetylation at the E-cadherin promoter region, knockdown of Bit1 enhanced TLE1 occupancy and reduced histone acetylation on the E-cadherin promoter. Importantly, ectopic Bit1 attenuated TLE1 induced E-cadherin suppression and increase in cell migration. Based on these findings, we propose a model (Fig 8) wherein Bit1 relieves E-cadherin repression in part through removal of TLE1 and its associated HDAC/PcG factors from the E-cadherin promoter and hence functions in maintenance of epithelial phenotype. During lung cancer progression and metastatic disease, loss or inactivation of Bit1 function may yield to heightened TLE1 repression of E-cadherin. Although the exact mechanism underlying the regulation of the TLE1 corepressor function by Bit1 remains to be examined, it may involve the transcriptional regulator protein AES, a Groucho related binding partner of TLE1. It is possible that Bit1, which is tethered on the outer mitochondrial membrane facing the cytoplasm [22], may interact with AES and such functional interaction impinges on TLE1 nuclear function. Alternatively, the Bit1 regulation of E-cadherin expression may involve other known EMT promoting signaling pathways. In particular, future mechanistic studies will focus on the role of FAK, which has been shown to interact with Bit1 [23], and ERK pathway, shown to be inhibited by Bit1 [6] in the Bit1/AES regulation of E-cadherin expression.

Tumor metastasis is a complex multistep process involving cancer cell migration and invasion of the extracellular matrix (ECM), adhesion with ECM components, intravasation through the blood vessel wall, survival in circulation, and ultimately lodging into secondary sites [12]. Effective inhibition of this process will likely require a multipronged approach that targets several steps concurrently. The dual function of Bit1 to inhibit anoikis resistance and EMT makes it a suitable therapeutic target in circumventing lung cancer aggressiveness. As discussed above, the dual role of Bit1 is possible due its inhibition of the TLE1 corepressor, a putative lung specific oncogene [24]. As a transcriptional repressor, TLE1 mediates gene regulatory function promoting both cell survival [1–4] and EMT [13] programs. Interestingly, the CtBP corepressor protein also co-ordinately regulates cell survival and EMT genetic programs, and is a target of several known tumor suppressors including HIPK2, Ink4a/Arf, and APC [25]. Like most other traditional transcriptional corepressors, TLE1 lacks a DNA-binding motif and is recruited to target gene promoters likely through interaction with DNA binding transcription factors. In the case of TLE1-mediated E-cadherin repression in NSCLC cells, Zeb1, an EMT promoting factor uniquely correlated with the loss of E-cadherin expression in human lung cancer cell lines [17], may, in part, underlie TLE1 recruitment to the E-cadherin promoter (Fig 8). Undoubtedly, identification of the critical components of the TLE1 corepressor complexes including other known E-cadherin transcriptional repressors and novel chromatin remodelling enzyme(s) will underscore the importance of TLE1 as a regulator of EMT in lung cancer.

In summary, we have uncovered a novel function of Bit1 in inhibiting cell motility and EMT in NSCLC. Mechanistically, Bit1 acts as a tumor migratory and EMT suppressor through maintenance of epithelial phenotype and induction of E-cadherin expression. Importantly, we show that Bit1 in conjunction with AES upregulates E-cadherin expression transcriptionally in part by targeting and inhibiting the TLE1 corepressor function. Our collective findings highlight the Bit1/AES/TLE1 pathway as an important determinant of lung cancer progression, and hence its downregulation and/or inactivation [9] may contribute to lung cancer aggressiveness and metastasis. By virtue of its ability to dually reverse EMT and anoikis resistance, the Bit1 signaling pathway may serve as an important platform in discovering novel therapeutic targets that will combat metastatic lung cancer.

## Supporting Information

S1 Fig The effect of alteration of Bit1 expression on growth of A549 and BEAS-2B cells. A. Stable control shRNA and Bit1 shRNA A549 cells were plated onto regular tissue culture plates and the growth of cells was quantified by MTT assay at the indicated time points. B. A549 cells treated with control or Bit1 siRNAs were subjected to MTT assay and their growth was quantified at the indicated time points. C. Stable control shRNA and Bit1 shRNA BEAS-2B cells were also subjected to MTT assay at the indicated time points. D. BEAS-2B cells treated with control or Bit1 siRNAs were subjected to MTT assay to quantify their growth rate at the indicated time points. E. A549 cells transfected with Bit1 mito, Bit1 cyto, or vector construct were subjected to MTT assay and their growth was assessed at the indicated time points.
(TIFF)

S2 Fig Knockdown of Zeb1 expression attenuates TLE1-mediated E-cadherin repression. A. and B. Stable control and TLE1 expressing pool of A549 cells were treated with control or Zeb1 siRNAs, and 48 hr later cells were subjected to immunoblotting with the indicated antibodies (A) and E-cadherin promoter luciferase assay (B). In B, * indicates p<0.05 by Student’s t test.
(TIFF)

S3 Fig Attenuation of Bit1-induced E-cadherin expression by TLE1 depends on Zeb1. A and B. Stable control and TLE1 expressing A549 cells were treated with control or Zeb1 siRNAs, and 24 h later cells were transfected with vector or Bit mito construct as indicated. Cells were then harvested and subjected to immunoblotting with the indicated antibodies (A). In parallel, cells were subjected to E-cadherin promoter luciferase assay (B). In B, * indicates p<0.05 by Student’s t test.
(TIFF)
