# Supplementary figures and images for: The Anoikis Effector Bit1 Inhibits EMT through Attenuation of TLE1-Mediated Repression of E-Cadherin in Lung Cancer Cells
Source: PLoS One. 2016 Sep 21;11(9):e0163228. doi: 10.1371/journal.pone.0163228 (PMC5031426; doi:10.1371/journal.pone.0163228)

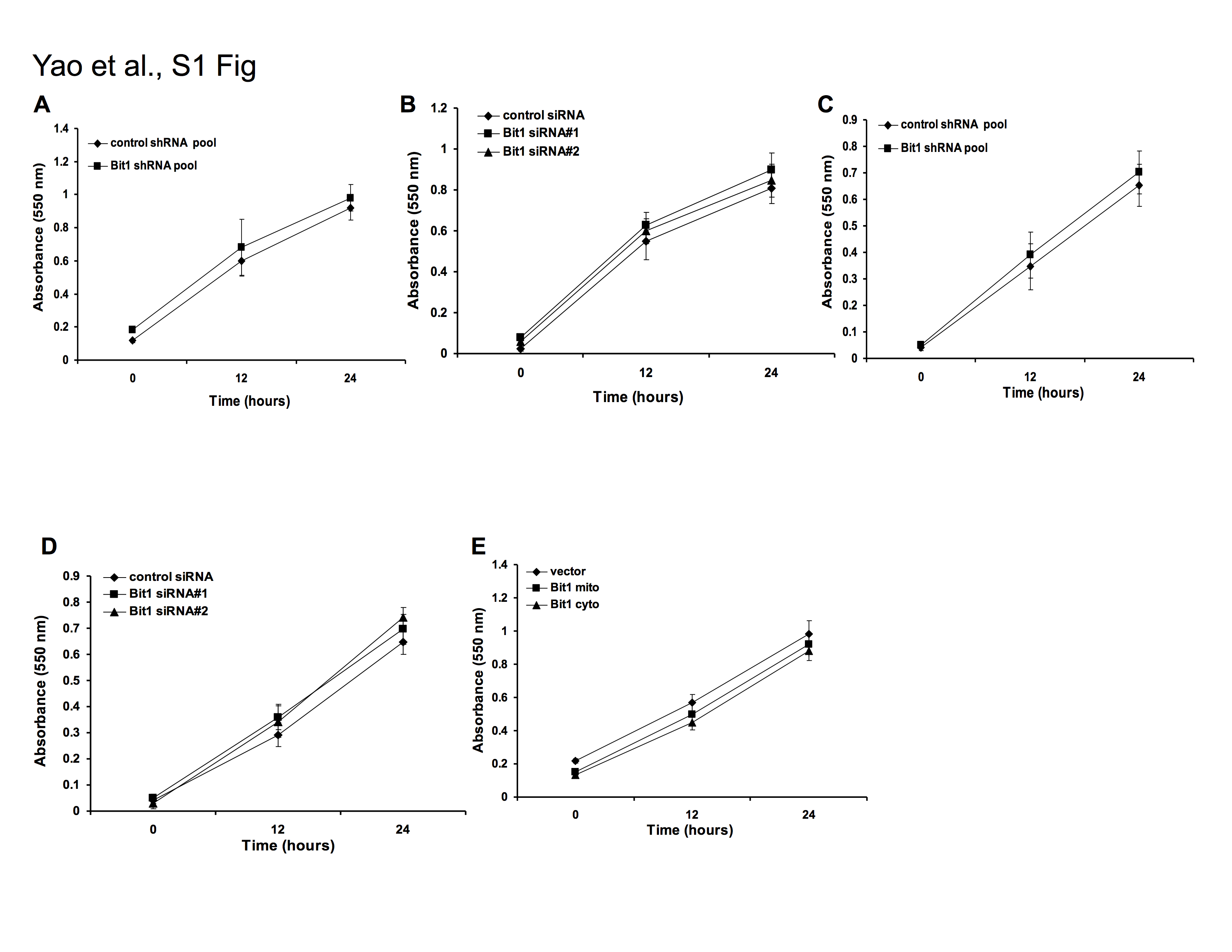

Supplement: S1 Fig — A. Stable control shRNA and Bit1 shRNA A549 cells were plated onto regular tissue culture plates and the growth of cells was quantified by MTT assay at the indicated time points. B. A549 cells treated with control or Bit1 siRNAs were subjected to MTT assay and their growth was quantified at the indicated time points. C. Stable control shRNA and Bit1 shRNA BEAS-2B cells were also subjected to MTT assay at the indicated time points. D. BEAS-2B cells treated with control or Bit1 siRNAs were subjected to MTT assay to quantify their growth rate at the indicated time points. E. A549 cells transfected with Bit1 mito, Bit1 cyto, or vector construct were subjected to MTT assay and their growth was assessed at the indicated time points. (TIFF) [file pone.0163228.s001.tiff]

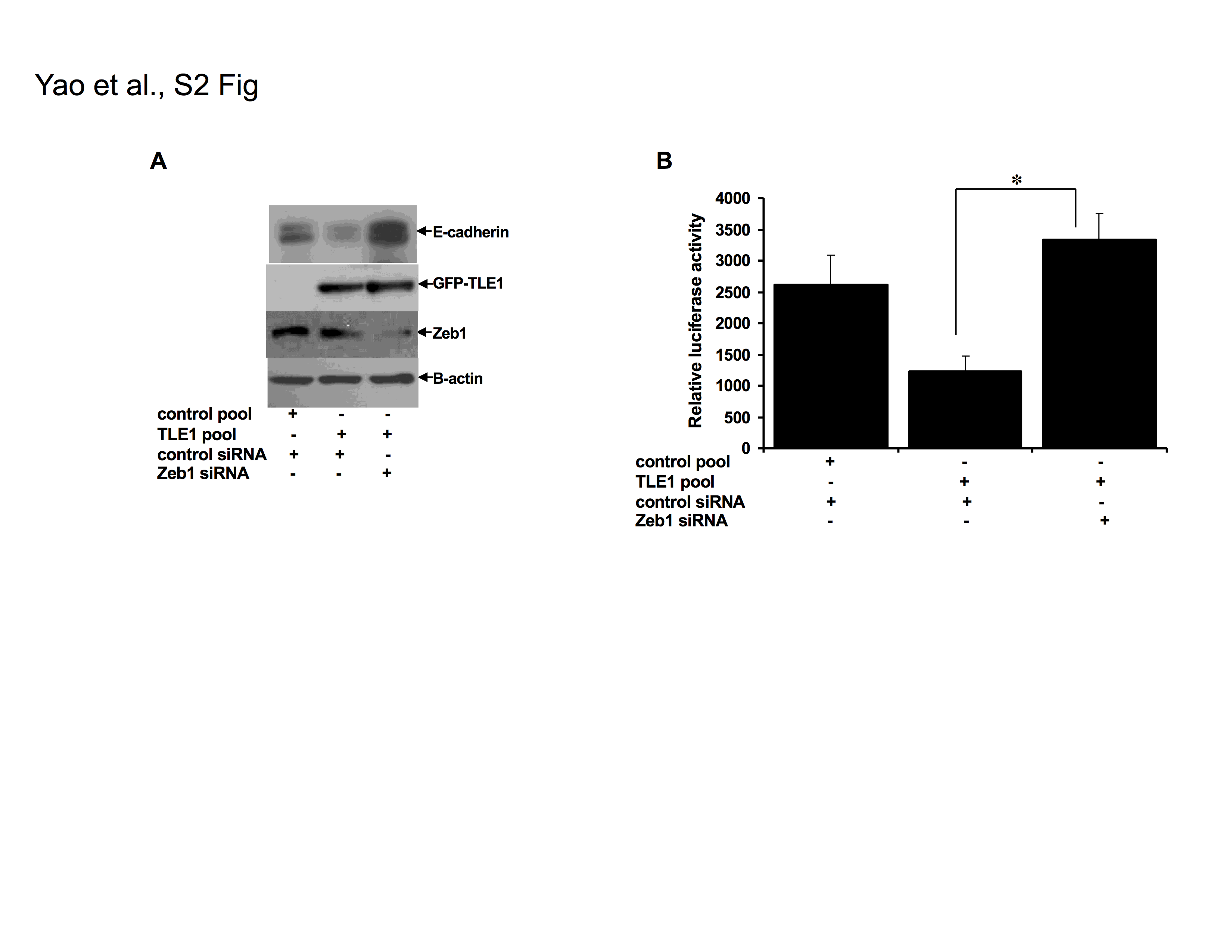

Supplement: S2 Fig — A. and B. Stable control and TLE1 expressing pool of A549 cells were treated with control or Zeb1 siRNAs, and 48 hr later cells were subjected to immunoblotting with the indicated antibodies (A) and E-cadherin promoter luciferase assay (B). In B, * indicates p<0.05 by Student’s t test. (TIFF) [file pone.0163228.s002.tiff]

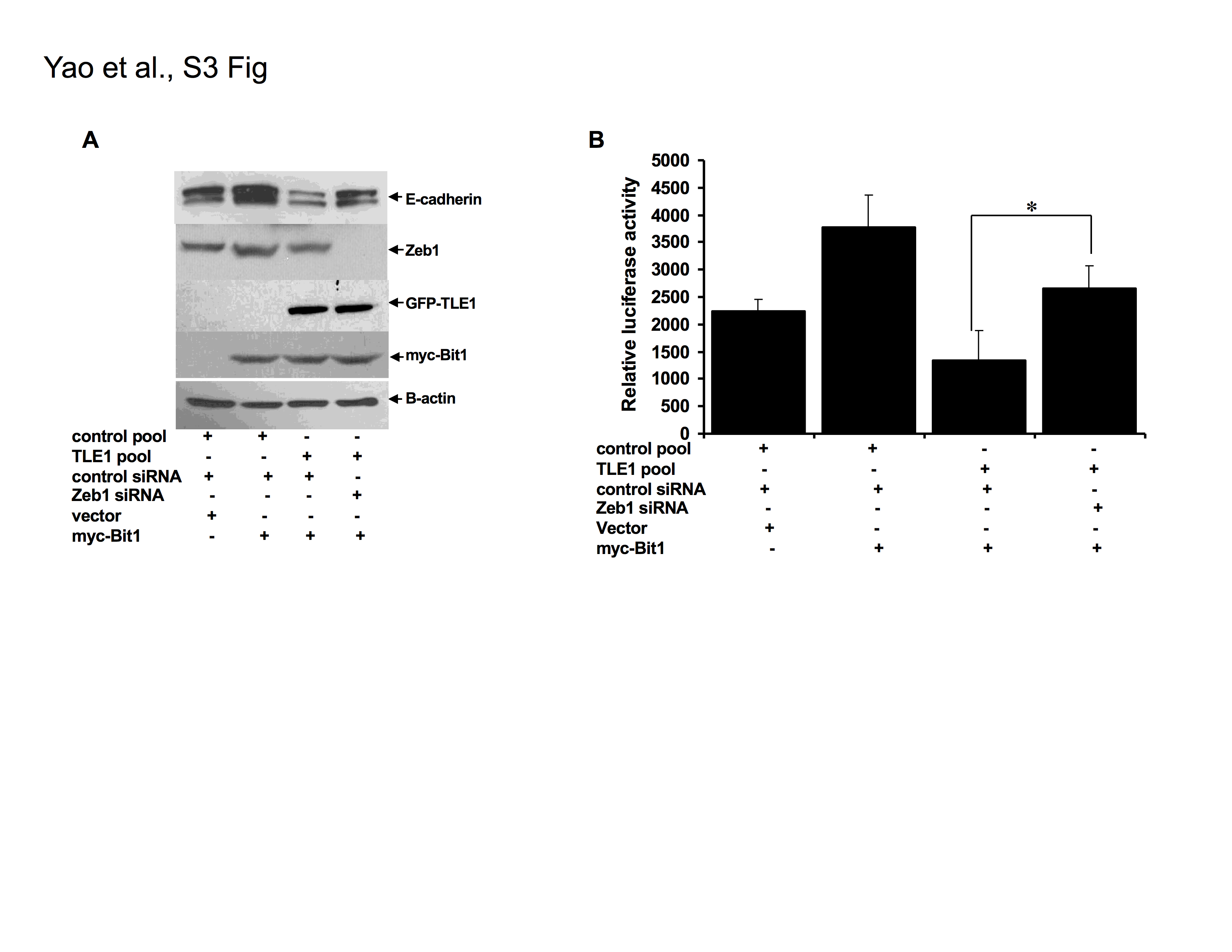

Supplement: S3 Fig — A and B. Stable control and TLE1 expressing A549 cells were treated with control or Zeb1 siRNAs, and 24 h later cells were transfected with vector or Bit mito construct as indicated. Cells were then harvested and subjected to immunoblotting with the indicated antibodies (A). In parallel, cells were subjected to E-cadherin promoter luciferase assay (B). In B, * indicates p<0.05 by Student’s t test. (TIFF) [file pone.0163228.s003.tiff]
